# Facial emotion recognition in adult with traumatic brain injury: A protocol for systematic review and meta-analysis

**DOI:** 10.1097/MD.0000000000021154

**Published:** 2020-07-17

**Authors:** XiaoGuang Lin, XueLing Zhang, QinQin Liu, PanWen Zhao, Hui Zhang, HongSheng Wang, ZhongQuan Yi

**Affiliations:** a Department of Neurology, Affiliated Suqian Hospital of Xuzhou Medical University, Suqian; b Department of Central Laboratory; c Department of Neurosurgery, Affiliated Yancheng School of Clinical Medicine, Nanjing Medical University, Yancheng, P.R. China.

**Keywords:** facial emotion recognition, meta-analysis, protocol, systematic review, traumatic brain injury

## Abstract

**Background:**

Traumatic brain injury (TBI) refers to head injuries that disrupt normal function of the brain. TBI commonly lead to a wide range of potential psychosocial functional deficits. Although psychosocial function after TBI is influenced by many factors, more and more evidence shows that social cognitive skills are critical contributors. Facial emotion recognition, one of the higher-level skills of social cognition, is the ability to perceive and recognize emotional states of others based on their facial expressions. Numerous studies have assessed facial emotion recognition performance in adult patients with TBI. However, there have been inconsistent findings. The aim of this study is to conduct a meta-analysis to characterize facial emotion recognition in adult patients with TBI.

**Methods:**

A systematic literature search will be performed for eligible studies published up to March 19, 2020 in three international databases (PubMed, Web of Science and Embase). The work such as article retrieval, screening, quality evaluation, data collection will be conducted by two independent researchers. Meta-analysis will be conducted using Stata 15.0 software.

**Results:**

This meta-analysis will provide a high-quality synthesis from existing evidence for facial emotion recognition in adult patients with TBI, and analyze the facial emotion recognition performance in different aspects (i.e., recognition of negative emotions or positive emotions or any specific basic emotion).

**Conclusions:**

This meta-analysis will provide evidence of facial emotion recognition performance in adult patients with TBI.

**INPLASY registration number:**

INPLASY202050109.

## 1 Introduction

Traumatic brain injury (TBI) refers to head injuries that disrupt normal function of the brain.^[1]^ These damages typically arise when there is a sudden acceleration–deceleration insult to the brain, such as during motor vehicle accidents, falls, sporting injuries, or assaults.^[2]^ Currently, TBI is a major cause of mortality and disability worldwide,^[3,4]^ with 10 million new cases annually.^[5]^ For survivors, more than 43% have experienced long-term disability.^[6]^ In addition, TBI commonly lead to a wide range of potential psychosocial functional deficits,^[7,8]^ which may result in a breakdown of social function for TBI, such as loss of employment, reduced social networks and disruption to intimate relationships.^[9–13]^

Although psychosocial function after TBI is influenced by many factors, more and more evidence shows that social cognitive skills are critical contributors.^[14–16]^ Social cognition can be defined as “the mental operations that underlie social interactions, including perceiving, interpreting, and generating responses to the intentions, dispositions, and behaviors of others.”^[17,18]^ One of the higher-level skills of social cognition is facial emotion recognition, which is the ability to perceive and recognize emotional states of others based on their facial expressions. Deficits in the recognition of basic emotions such as anger, disgust, fear, sad, happy, and surprise can lead to misinterpretation of social cues that guide normal behavior, which make contribution to difficulties with social conduct. In general, accurate facial emotion recognition is deemed to be necessary for effective interpersonal functioning and communication.^[19]^

Recently, a number of studies have assessed the facial emotion recognition deficits in adult patients with TBI.^[20–23]^ However, there have been inconsistent findings regarding the specific emotion recognition deficits in TBI. For example, Wearne et al^[24]^ found that compared to healthy controls (HC), adult patients with TBI have overall accuracy in recognizing emotion, specifically for happy and sad emotions, while Byom et al^[25]^ found no difference between TBI patients and HC in happy, but significant difference for expressions of sad. Besides, the sample sizes in these studies were small and there was significant variability in the magnitude of group differences between adult patients with TBI and HC. A meta-analysis is helpful to increase the statistical power and to clarify findings of inconsistent findings in individual studies. So in this study, we will conduct a meta-analysis to investigate the magnitude of emotion recognition deficits in adult patients with TBI in comparison to HC. In addition, we will conduct subgroup meta-analyses to establish whether difficulties in recognition of negative emotions (anger, disgust, fear, and sad) or positive emotions (happy and surprise) or any specific basic emotion (i.e., anger or happy) can be a more distinctive feature of TBI. Furthermore, meta-regression analyses will be performed to examine the effects of potential confounders on emotion recognition deficits, such as age, gender, education level, and disease duration. Our meta-analysis will be helpful to understand the patterns of emotion recognition function in adult patients with TBI, which may be important for identification of targets for affect recognition interventions and developing useful training intervention programs.

## 2 Methods

### 2.1 Study registration

This systematic review is registered on INPLASY (INPLASY202050109). It has been reported according to the guidelines of the Preferred Reporting Items for Systematic Reviews and Meta-Analyses Protocols (PRISMA-P) statement.^[26]^ Ethical approval is not required because the data used in this paper are from published studies without the involvement of individual or animals experiments.

### 2.2 Criteria of selection for study

#### 2.2.1 Criteria for inclusion

1. The study should be published as a primary peer-reviewed research article in English.
2. The onset age of TBI patients was not <18 years old.
3. The study had to examine facial emotion recognition abilities.
4. Sufficient data to calculate effect sizes and standard errors of the facial emotion recognition measure were reported.
5. A matched HC group had to be included.

#### 2.2.2 Criteria for exclusion

1. The onset age of TBI patients was <18 years old.
2. The study with the patient samples was overlapped with another one with a larger sample size.
3. The study lacked an HC group.
4. A study with a sample size under 10 will be excluded to ensure the reliability of the outcome.
5. The publication was not an original type, such as research protocols, letters, conference abstracts, reviews, and editorials.

#### 2.2.3 Type of outcome measure

Primary outcomes will include the facial emotion recognition measure used and the data used for calculating the effect sizes and standard errors of the facial emotion recognition measure. Additional outcomes will include the Glasgow Coma Scale scores (GCS) and other questionnaire of clinical symptoms of TBI.

### 2.3 Data sources

#### 2.3.1 Electronic searches

Electronic databases, including PubMed, Web of Science and Embase, have been searched from inception to May 19, 2020 with no restriction of publication dates. In addition, other resources have been searched manually, such as the references of all included studies.

#### 2.3.2 Search strategy

Search terms are related to TBI and facial emotion recognition. Related Medical Subject Heading (MeSH) terms and synonyms in various combinations are used as search strategies. The terms to be used in relation to the disease include “traumatic brain injury,” “brain trauma,” “closed head injury,” “head injury,” “head trauma,” “prefrontal cortex damage,” and “TBI.” The terms to be used in relation to the facial emotion recognition include “facial emotion recognition,” “emotion recognition,” “social cognition,” and “emotion.” The search strategies are presented in Table 1.

**Table 1:**
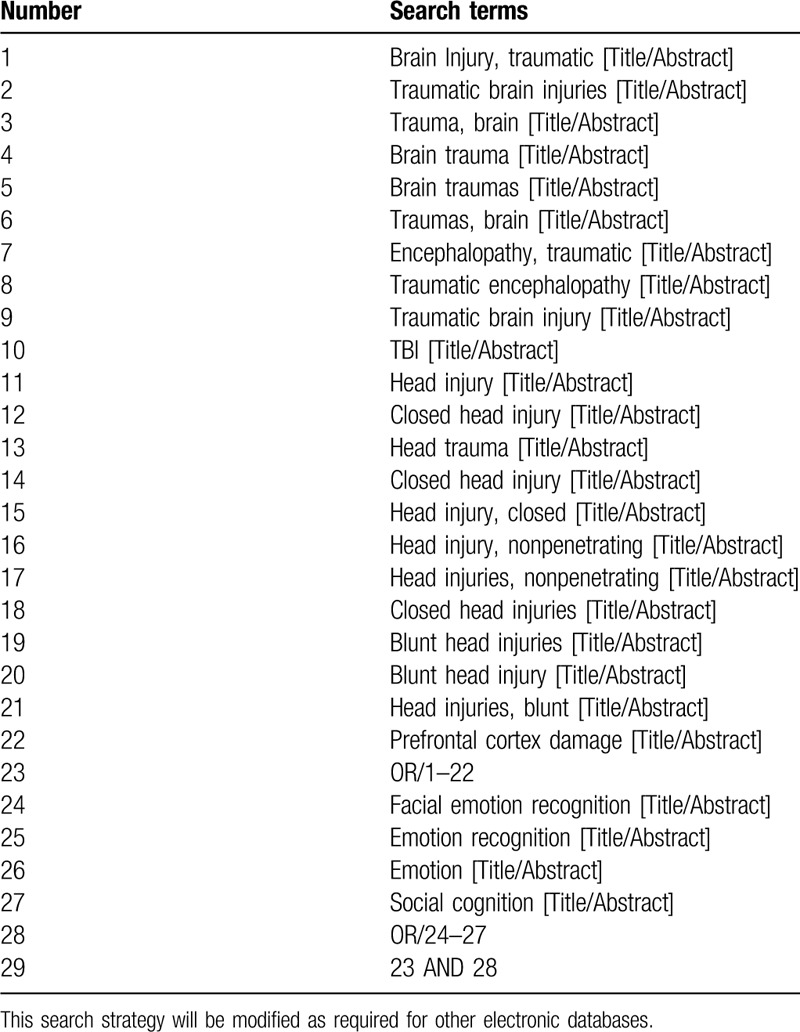
Represents the search strategy for PubMed database.

### 2.4 Data collection and analysis

#### 2.4.1 Selection of studies

**Figure 1:**
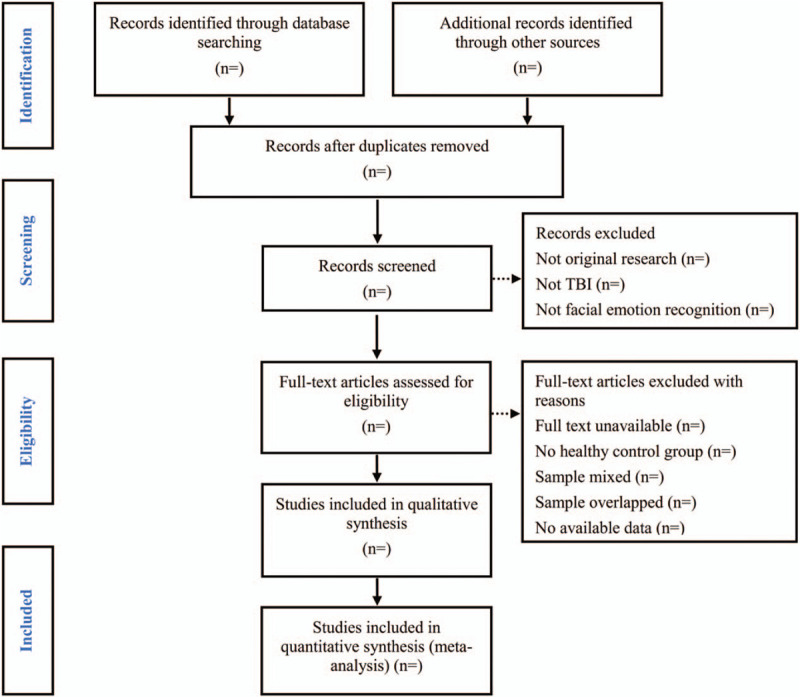
Flow diagram of studies search and selection.

We will use the PRISMA flow chart to show the process of selecting literature for the entire study (Fig. 1). We will manage all literatures by using EndNote software, V.X7 (United States). Two investigators (XGL and XLZ) will independently review and screen the literature based on predetermined inclusion and exclusion criteria. If there is a disagreement between the two investigators, discussion will be held with the third investigator (ZQY) for arbitration.

#### 2.4.2 Assessment of quality in included studies

We will use the Newcastle-Ottawa Quality Assessment Scale (NOS) to assess the quality of all included studies.^[27]^

#### 2.4.3 Data extraction and management

Two investigators (QQL and PWZ) will independently extract data. The information will include first author, publication year and title, TBI diagnosis criteria, inclusion/exclusion criteria, number of groups, number of participants, patients’ age, sex, education level, disease duration, TBI stage, HC’ age, sex, education level, the facial emotion recognition measure used and the data used for calculating the effect sizes and standard errors of the facial emotion recognition measure and adverse events. Any discrepancies in the data will be reviewed by another researcher (ZQY).

### 2.5 Data synthesis and statistical analysis

#### 2.5.1 Dealing with missing data

For included studies in which there are missing data or the analysis process is unclear, the associated risk of bias will be fully considered. The authors will be contacted via email about information that is not available in the study. If data are still insufficient after contacting the author, it will be analyzed using the available data.

#### 2.5.2 Data synthesis

Data analysis will be performed using Stata 15.0 software. The total emotion labeling score and separate effect sizes for six basic emotions (anger, fear, disgust, sad, happy, and surprise) were calculated. A separate negative emotion recognition score was obtained by calculating the pooled effect size and standard error of anger, disgust, sad, and fear recognition. Similarity, a separate positive emotion recognition score was obtained by calculating the pooled effect size and standard error of happy and surprise recognition. Effect sizes <0.5 were considered small, between 0.5 and 0.8 moderate, and >0.8 large. When appropriate, data will be pooled across studies for meta-analysis using fixed or random effect models. When quantitative synthesis is not appropriate due to heterogeneity, we will offer summary tables of study characteristics and outcome measures and do a narrative synthesis.

#### 2.5.3 Assessment of heterogeneity

We will assess the heterogeneity by the *I*^2^ statistic base on a standard linear hypothesis with *I*^2^ < 50 indicating low heterogeneity. The fixed-effects model will be applied to homogeneous data (*I*^2^ value < 50%), and if *I*^2^ value ≥50% (*P*-value < .10), the random-effects model will be applied.

#### 2.5.4 Assessment of publication bias

If the analysis includes ≥10 studies in meta-analysis, a funnel plot will be used to detect publication bias.

#### 2.5.5 Sensitivity analysis

To assess the stability of the results, a sensitivity analysis was performed by repeating the same analyses by consecutively removing one study at a time.

#### 2.5.6 Subgroup analysis

Subgroup analysis will be performed in different aspects of facial emotion recognition (including negative emotion recognition, positive emotion recognition, and six basic emotion recognition) and in clinical subtypes (such as mild TBI patients and moderate to severe TBI patients).

#### 2.5.7 Meta-regression analysis

Meta-regression analyses will be conducted for variables including the age, gender, education level, GSC score and disease duration, with a random-effects model using the restricted-information maximum likelihood method with the significance level set at *P* < .05.

## 3 Discussion

To the best of our knowledge, this is the first research protocol to examine facial emotion recognition in adult with TBI. In this systematic review, we will assess the quality of evidence with NOS tool, and two independent reviewers will conduct the study selection, data extraction, and methodological quality assessment, whereas any disagreements will be settled down with a third reviewer through discussion. This study will be helpful to understand the patterns of emotion recognition function in adult patients with TBI, which may be important for identification of targets for affect recognition interventions and developing useful training intervention programs.

## Author contributions

**Conceptualization:** XiaoGuang Lin, XueLing Zhang.

**Data curation:** QinQin Liu, PanWen Zhao.

**Investigation:** XiaoGuang Lin, QinQin Liu.

**Methodology:** XueLing Zhang, Hui Zhang.

**Supervision:** XiaoGuang Lin, HongSheng Wang.

**Validation:** ZhongQuan Yi.

**Writing – original draft:** XiaoGuang Lin.

**Writing – review & editing:** ZhongQuan Yi, HongSheng Wang.
